# Inferring structural connectivity using Ising couplings in models of neuronal networks

**DOI:** 10.1038/s41598-017-05462-2

**Published:** 2017-08-15

**Authors:** Balasundaram Kadirvelu, Yoshikatsu Hayashi, Slawomir J. Nasuto

**Affiliations:** Brain Embodiment Lab, Biomedical Engineering, School of Biological Sciences, University of Reading, Reading, United Kingdom

## Abstract

Functional connectivity metrics have been widely used to infer the underlying structural connectivity in neuronal networks. Maximum entropy based Ising models have been suggested to discount the effect of indirect interactions and give good results in inferring the true anatomical connections. However, no benchmarking is currently available to assess the performance of Ising couplings against other functional connectivity metrics in the microscopic scale of neuronal networks through a wide set of network conditions and network structures. In this paper, we study the performance of the Ising model couplings to infer the synaptic connectivity in *in silico* networks of neurons and compare its performance against partial and cross-correlations for different correlation levels, firing rates, network sizes, network densities, and topologies. Our results show that the relative performance amongst the three functional connectivity metrics depends primarily on the network correlation levels. Ising couplings detected the most structural links at very weak network correlation levels, and partial correlations outperformed Ising couplings and cross-correlations at strong correlation levels. The result was consistent across varying firing rates, network sizes, and topologies. The findings of this paper serve as a guide in choosing the right functional connectivity tool to reconstruct the structural connectivity.

## Introduction

Recent developments have made it possible to record the simultaneous spiking activity of hundreds of neurons^1,2^. Functional connectivity is a statistical description of the mutual dependencies observed in multi-neuronal spiking activity^3^. Functional connectivity has a non-trivial relationship with the underlying anatomical architecture of the neuronal circuits^4^. The ability to reconstruct the underlying structural connectivity from the functional connectivity remains an important open question^5,8^.

The most common functional connectivity measure used in the study of neuronal activity is cross-correlation^9^. However, the usefulness of cross-correlation in inferring the structural connectivity in a neuronal network is limited. Due to the fact that each pair of nodes is considered independently in calculating the cross-correlations, it is not possible to determine if the correlated activity of a neuron pair is the result of direct or indirect connection between them, or the result of a common input^10,11^. Partial correlation overcomes this limitation by removing the effects of the activity of all other neurons (assuming that the effects are additive) while assessing the relationship between two spike trains^12^. A recent study^13^ has confirmed that partial correlation outperforms cross-correlation in inferring the structural connectivity in simulated networks.

Another approach in obtaining the functional connectivity of a neuronal network is to tune the parameters of a statistical model so that the probability distribution of the spike data generated by that model agrees with the probability distribution of the spike data recorded from the neuronal network^14^. Then the parameters of the model can be considered to represent the functional connections between the neurons in the network. Shlens & co-workers^15^ and Schneidman & co-workers^16^ observed that the probability distribution of the binary second-order maximum entropy model was able to explain around 90% of the spatial correlation structure of the spike data recorded from groups of neurons. This conclusion has also been later reported by other research groups for both *in vivo*^17^ and *in vitro*^18^ recordings. The probability distribution of a second-order maximum entropy model is identical to the Gibbs equilibrium distribution (as given by equation (1)) of the Ising model, widely used in statistical physics^14,16^. Thus, the coupling parameter of the Ising model lends itself as an alternative measure of functional connectivity. The probability distribution of the Ising model is given by the following equation:

1 $$P(\sigma )=\frac{1}{Z}\exp [-\sum _{{\rm{i}}}{{\rm{h}}}_{{\rm{i}}}{{\rm{\sigma }}}_{{\rm{i}}}-0.5\sum _{{\rm{i}},{\rm{j}}}{{\rm{J}}}_{{\rm{ij}}}{{\rm{\sigma }}}_{{\rm{i}}}{{\rm{\sigma }}}_{{\rm{j}}}]$$

where $\sigma =\{{\sigma }_{i}\}$ and σ^*i*^ = ±1 represents the presence or absence of a spike of a neuron *i* in a time window. The external field parameter *h*_*i*_ and the coupling parameter *J*_*ij*_ are the parameters of the Ising model. *Z* is the normalizing denominator and is called the partition function.

Studies^16,17,19,20^ suggest that the Ising coupling parameters can remove the effects of the indirect interactions and account only for the direct interactions rendering Ising coupling parameter as a robust indicator of the underlying structural connectivity in the network. Watanabe *et al*.^21^ show that the Ising couplings from a resting state fMRI data reflect the anatomical connections of the brain more accurately than partial correlation and other functional connectivity measures. To the best of our knowledge, no comparison has been carried out between Ising couplings and partial correlations in the microscopic scale of neuronal networks, testing through a wide set of network conditions and network structures. In our paper, we set out to systematically study the relationship between Ising couplings and the underlying structural connections and contrast it to partial and cross-correlations, in *in silico* neuronal networks. We also address the question of how firing rate, network correlation, network size, and topology affect the performance of the Ising couplings to unravel the true anatomical structure of neuronal networks.

We use *in silico* networks in our study as the structural connections are known and different network conditions can be easily controlled in simulations. It is difficult to evaluate the performance of a functional connectivity tool to infer the underlying synaptic connectivity in *in vivo* or *in vitro* neuronal networks as the real anatomical connectivity in those networks is not known fully^5^. We evaluate Ising couplings against partial and cross-correlations in scale-free, modular small-world and random network topologies of *in silico* networks, as studies^22^ suggest that the structural connectivity in neuronal networks exhibits features of complex networks. Studies support the existence of scale-free network connectivity in primary cortical cultures^23^ and developing hippocampal networks^24^. Analysis of the activity of cultured neurons during maturation suggests an evolution of the network structure from a random topology to a small-world topology^25^. We also evaluate the three functional connectivity metrics for different firing rates and correlation levels in networks of various sizes and network densities, as literature^6,7^ indicates that the activity of neuronal networks is characterized by such factors. In summary, in this work, we systematically study the predictability of the underlying structural connections by Ising couplings, in comparison to partial and cross-correlations, in *in silico* neuronal networks and how the predictability is affected by different network conditions.

The paper is organised as follows. A detailed account of the *in silico* networks and the simulation of the network of neurons is presented in the methods section. The same section also describes the calculation of the Ising parameters, partial and cross-correlations from the spike trains. The results of the systematic comparison between Ising parameters, partial correlations and cross-correlations are presented and evaluated in the results and discussions sections respectively.

## Methods

### Simulation Network

The simulation network consisted of *N* Izhikevich spiking model neurons^26^. Izhikevich model was chosen for its computational efficiency and its capability to generate several firing patterns based on four parameters^27^. All the neurons were modeled as excitatory neurons. To achieve heterogeneity in the spiking dynamics, the neurons were modeled using the parameters (a, b) = (0.02, 0.2) and (c, d) = (−65, 8) + (15, −6)*r*^2^, where r is a uniformly distributed random variable in the interval (0, 1). The case of *r* = 0 corresponds to a regular spiking neuron model, and *r* = 1 corresponds to a chattering neuron model. *r*^2^ was used to bias the distribution towards regular spiking neurons^28^.

Inspired by Rocha *et al*.^29^, we modeled the input current, *I*_*i*_, to each neuron *i* using the equation

2 $${I}_{i}={I}_{i}^{base}+[\mathrm{(1}-CF)\ast {I}_{i}^{thalamic}+CF\ast {I}_{i}^{synaptic}]$$

The total input current *I*_*i*_ to each neuron *i* consisted of 3 components: ${I}_{i}^{base}$ was a constant input with an additive Gaussian noise of zero mean and unit variance which influenced the average firing rate of the neuron. ${I}_{i}^{thalamic}$ was a noisy random input which was given by a Gaussian variable multiplied by a constant and was uncorrelated for any two neurons. ${I}_{i}^{synaptic}$ of a neuron *i* was the sum of the synaptic inputs from the presynaptic neurons connected to it. *CF* was a global control factor variable (0 ≤ CF ≤ 1) which determined the amount of correlation between the firing of the neurons in the networks by controlling the relative contribution of *I*^*thalamic*^ and *I*^*synaptic*^. When *CF* = 0, the contribution of *I*^*synaptic*^ to total input became zero and the input of a neuron was influenced by the noisy *I*^*thalamic*^, and hence the firing between the neurons was less correlated. When *CF* = 1, the contribution of the *I*^*thalamic*^ component became zero. The firing of a neuron increased the *I*^*synaptic*^ of its postsynaptic neuron and hence the postsynaptic neuron had higher chances of firing together with the presynaptic neuron. Thus, neurons spiked together more when *CF* = 1. The network of Izhikevich neurons was simulated for different values of average firing rates and correlation levels by adjusting the values of *I*^*base*^ and *CF*.

The connectivity between the neurons was given by the adjacency matrix A = (*w*_*ij*_). The firing of the *j* th neuron affected the voltage of the *i* th neuron by an amount *w*_*ij*_ multiplied by $CF$. The strengths of the links (non-zero *w*_*ij*_ in the adjacency matrix) were distributed normally with a mean of 0.6 and a standard deviation of 0.13 and were limited to the interval [0.21, 0.99]. Self-loops were not allowed. The adjacency matrix for each simulated topology (scale-free, small-world, and random networks) was generated using the corresponding topology generation algorithms. Scale-free (SF) topology was generated using directed preferential attachment model for network growth^30^. Brain connectivity toolbox^31^ was used to generate modular small-world (SW) topology with a specified number of fully connected modules connected via randomly distributed inter-module connections. Erdos-Renyi (ER) random networks were generated with a fixed connection probability between all pairs of neurons. For all topologies, the total number of links in the network was fixed at 20% of the total possible links (which is *N* * (*N* − 1)) as studies^13,32,33^ suggest that on an average each neuron is connected to 10% to 30% of the other neurons in *in vitro* cultures.

### Data from the simulation network

The neuronal network was then simulated for a length of time to capture the spike train data. When the voltage of a neuron reached a threshold (which was a dynamic value, depending on the parameters of the neuron), a spike was initiated. The time of the spike and the number of the neuron which spiked was recorded to generate the spike train data of the simulated neuronal network. The duration of the simulation (which was set to 10 minutes) was then split into many time bins of equal width 10 ms considering the spike transmission delay in the network. The state of a neuron *i* in a time bin was represented by ${\sigma }_{i}$, and it took a value of +1 or −1 corresponding to the presence or absence of spikes in that time bin. The average firing rate ${\langle {\sigma }_{i}\rangle }_{data}$ of a neuron *i* and the average pairwise joint firing rate ${\langle {\sigma }_{i}{\sigma }_{j}\rangle }_{data}$ for a pair of neurons *i* and *j* were calculated using the following equations:

3 $${\langle {\sigma }_{i}\rangle }_{data}=\frac{1}{B}\sum _{b\mathrm{=1}}^{B}{\sigma }_{i}^{b},$$

4 $${\langle {\sigma }_{i}{\sigma }_{j}\rangle }_{data}=\frac{1}{B}\sum _{b\mathrm{=1}}^{B}{\sigma }_{i}^{b}\mathrm{.}{\sigma }_{j}^{b},$$

where the angle brackets indicate time averaging, *B* was the total number of time bins for the duration of the simulation and ${\sigma }_{i}^{b}$ was the state of the neuron *i* in a particular time bin *b*^18^. The covariance $Co{v}_{ij}$ between the firing of two neurons *i* and *j* was defined as $Co{v}_{ij}={\langle {\sigma }_{i}{\sigma }_{j}\rangle }_{data}-{\langle {\sigma }_{i}\rangle }_{data}\,\cdot \,{\langle {\sigma }_{j}\rangle }_{data}$. And, the correlation coefficient between the spike trains of the neurons *i* and *j* was calculated as ${\rho }_{ij}=\frac{Co{v}_{ij}}{{s}_{i}{s}_{j}}$ where ${s}_{i}$ was the standard deviation of firing activity ${\sigma }_{i}$ of the neuron *i*. The mean network correlation *ρ* was calculated as the average of the correlation coefficient between all pairs of neurons.

### Calculation of Ising parameters

We calculated the parameters *h*_*i*_ and ${J}_{ij}$ of the Ising model using Boltzmann learning^34^, which is a gradient descent algorithm. We started with an initial value for the parameters *h*_*i*_ and ${J}_{ij}$ and adjusted them iteratively according to equations (5) and (6) till the first and second order moments of the Ising model (${\langle {\sigma }_{i}\rangle }_{model}$ and ${\langle {\sigma }_{i}{\sigma }_{j}\rangle }_{model}$) agreed with the estimates obtained from the simulation data (${\langle {\sigma }_{i}\rangle }_{data}$ and ${\langle {\sigma }_{i}{\sigma }_{j}\rangle }_{data}$) within the desired accuracy.

5 $${h}_{i}^{new}={h}_{i}^{old}+\alpha \mathrm{.}({\langle {\sigma }_{i}\rangle }_{data}-{\langle {\sigma }_{i}\rangle }_{model})$$

6 $${J}_{ij}^{new}={J}_{ij}^{old}+\alpha \mathrm{.}({\langle {\sigma }_{i}{\sigma }_{j}\rangle }_{data}-{\langle {\sigma }_{i}{\sigma }_{j}\rangle }_{model})$$

where *α* is the learning rate, and it was kept less than 1 to get a smoother convergence.

As can be seen from the equations, the Ising model moments(〈*σ*_*i*_〉_*model*_ and 〈*σ*_*i*_*σ*_*j*_〉_*model*_) need to be calculated for each gradient descent iteration and is a computationally intensive task. We adopted the method found in Yeh *et al*.^35^ for computing the 〈*σ*_*i*_〉_*model*_ and 〈*σ*_*i*_*σ*_*j*_〉_*model*_ from the Ising parameters *h*_*i*_ and *J*_*ij*_. The exact method of calculating the Ising model moments has a computational complexity of the order ${\mathscr{O}}{\mathrm{(2}}^{N})$ and is possible only for small *N* (<20). For larger *N*, we used Monte Carlo sampling based on Metropolis-Hastings simulation which has a complexity of ${\mathscr{O}}(Iteration\,Number)$. A very large number of iterations of the order of 10^7^ was used in our simulation.

### Calculation of cross-correlation and partial correlation

Cross-correlation can be interpreted as the probability of a neuron (called the target neuron) spiking at a time $(t+\tau )$ conditioned on another reference neuron spiking at a time *t* where *τ* is called the time lag^13,36^. Let *x* and *y* be the spike trains of the reference  and the target  neurons, respectively. The cross-correlation function *C*_*xy*_(*τ*) is defined as follows^13^:

7 $${C}_{xy}(\tau )=\frac{1}{\sqrt{{N}_{x}{N}_{y}}}\sum _{s\mathrm{=1}}^{{N}_{x}}\,\sum _{{t}_{i}=(\tau -\frac{{\rm{\Delta }}\tau }{2})}^{(\tau +\frac{{\rm{\Delta }}\tau }{2})}x({t}_{s})y({t}_{s}-{t}_{i})$$

where *N*_*x*_ and *N*_*y*_ are the total number of spikes in the spike trains *x* and *y*, respectively, and ${t}_{s}$ is the timing of a spike in the spike train *x*. The cross-correlation function is symmetric. That is, if we compute the cross-correlation function keeping *x* as reference and *y* as the target and then compute cross-correlation function keeping *y* as reference and *x* as the target, we will get the same function but just reversed in time.

8 $${C}_{xy}(\tau )={C}_{yx}(-\tau )$$

The cross-correlation matrix (CCM) is an N × N matrix. The (i, j)-th element of the CCM (*CC*_*ij*_) corresponds to the maximum amplitude of the cross-correlation function for the neuron pair (i, j). Because of equation (8), the CCM matrix is symmetric in nature, i.e. CCM(i, j) = CCM(j, i). Cross-correlation fails to distinguish between direct and indirect connections as it is calculated pairwise for each pair without any consideration of the influence of the other elements of the network on the activity of the pair of neurons.

Partial correlation approach attempts to solve this problem by removing the linear contribution of other neurons in the population when calculating the dependence for a pair of neurons. Consider *x *and *y* as two neurons in a population P of neurons. The partialised cross-spectrum ${S}_{xy|P}$ between neurons *x* and *y* is obtained as follows^10,37^:

9 $${S}_{xy|P}={S}_{xy}-({S}_{xP}\,{S}_{PP}^{-1}\,{S}_{yP})$$

where *S*_*xy*_ is the full cross spectrum between the neurons *x* and *y*, *S*_*xP*_ (*S*_*yP*_) corresponds to the cross spectrum between the neuron *x* (*y*) and the population *P* and *S*_*PP*_ is the cross spectrum between the rest of the neurons in the population P. The partial correlation function is given by a scaled version of the inverse Fourier transform of ${S}_{xy|P}$. A good reference for the calculation of partial correlation can be found at Poli *et al*.^13^. Similar to CCM, partial correlation matrix (PCM) is a N × N symmetric matrix and the (i, j)-th element in the PCM ($P{C}_{ij}$) corresponds to the maximum amplitude of the partial correlation function for the neuron pair (i, j).

We computed cross-correlation and partial correlation matrices (CCM and PCM) using ToolConnect^37^, an open-source toolbox.

### Evaluation of functional connectivity matrices

The structural connectivity matrix (also called the adjacency matrix) is a directional and sparsely connected (i.e. connectivity defined only between specific pairs of neurons) binary matrix. The functional connectivity matrices (Ising coupling J matrix, CCM, PCM) are non-directional and generally, all-to-all connected matrices. For meaningful comparison between the structural and functional connectivity matrices, both matrices have to be reduced to a sparse, binary and non-directional form, through thresholding, binarising, and symmetrising^31^. Each functional connectivity matrix was thresholded based on the absolute values of its elements and then binarised to convert it to a sparsely connected matrix. The absolute value of the elements was considered for the thresholding of the functional connectivity matrices because negative values can occur in functional connectivity matrices (and may indicate inhibitory links in the structural topology). The structural connectivity matrix was symmetrised and converted to a non-directional matrix. We then compared the symmetrised structural connectivity matrix (SCM) against the thresholded and binarised functional connectivity matrices (FCM) for different threshold levels. We recorded the results of the comparison using the metrics of true positives (TP), false positives (FP), true negatives (TN) and false negatives (FN). If a non-zero value in the FCM corresponds to a non-zero value in the SCM, it is recorded as a TP. If a zero value in the FCM corresponds to a zero value in the SCM, it is recorded as a TN. If a zero value in the FCM corresponds to a non-zero value in the SCM, it is called an FN. If a non-zero value in the FCM corresponds to a zero value in the SCM, it is called as an FP.

We assessed the performance of the functional connectivity metrics to uncover the underlying structural connectivity by the amount of match between the SCM and the FCM for different threshold levels using the standard receiver operating characteristic (ROC) curve analysis. The ROC is a standard method to study the performance of a binary classifier as the classification threshold is varied^38^. The ROC curve is the plot of the relationship between the true positive ratio (TPR) and the false positive ratio(FPR) for different threshold levels. The TPR is defined as the ratio of the number of links in the FCM that match the existing links in SCM to the total number of links in the SCM. FPR is defined as the ratio of the links in FCM that do not match the links in SCM to the total number of zeros in the SCM. TPR and FPR are given by the following equations:

10 $$TPR=\frac{TP}{P}=\frac{TP}{(TP+FN)}$$

11 $$FPR=\frac{FP}{N}=\frac{FP}{(TN+FP)}$$

We then summarized the performance of the ROC curve in a single number using the common approach of calculating the area under the ROC curve (abbreviated as AUC)^38^. The value of the AUC varies between 0 and 1 as the ROC curve covers a portion of the area under unit square (both TRP and FPR vary from 0 to 1). A random classifier has a ROC curve along the diagonal line joining (0, 0) and (1, 1) and an AUC value of 0.5. A perfect classifier has a ROC curve that hugs the upper left corner of the plot and an AUC value of 1.0. The closer the value of AUC is to 1, the better is the classifier.

## Results

We compared the relationship between the functional connectivity matrices (Ising couplings, cross-correlations, and partial correlations) and the structural connectivity for different simulated network sizes, topologies, firing rates and network correlation levels.

### Effect of mean network correlation

**Figure 1:**
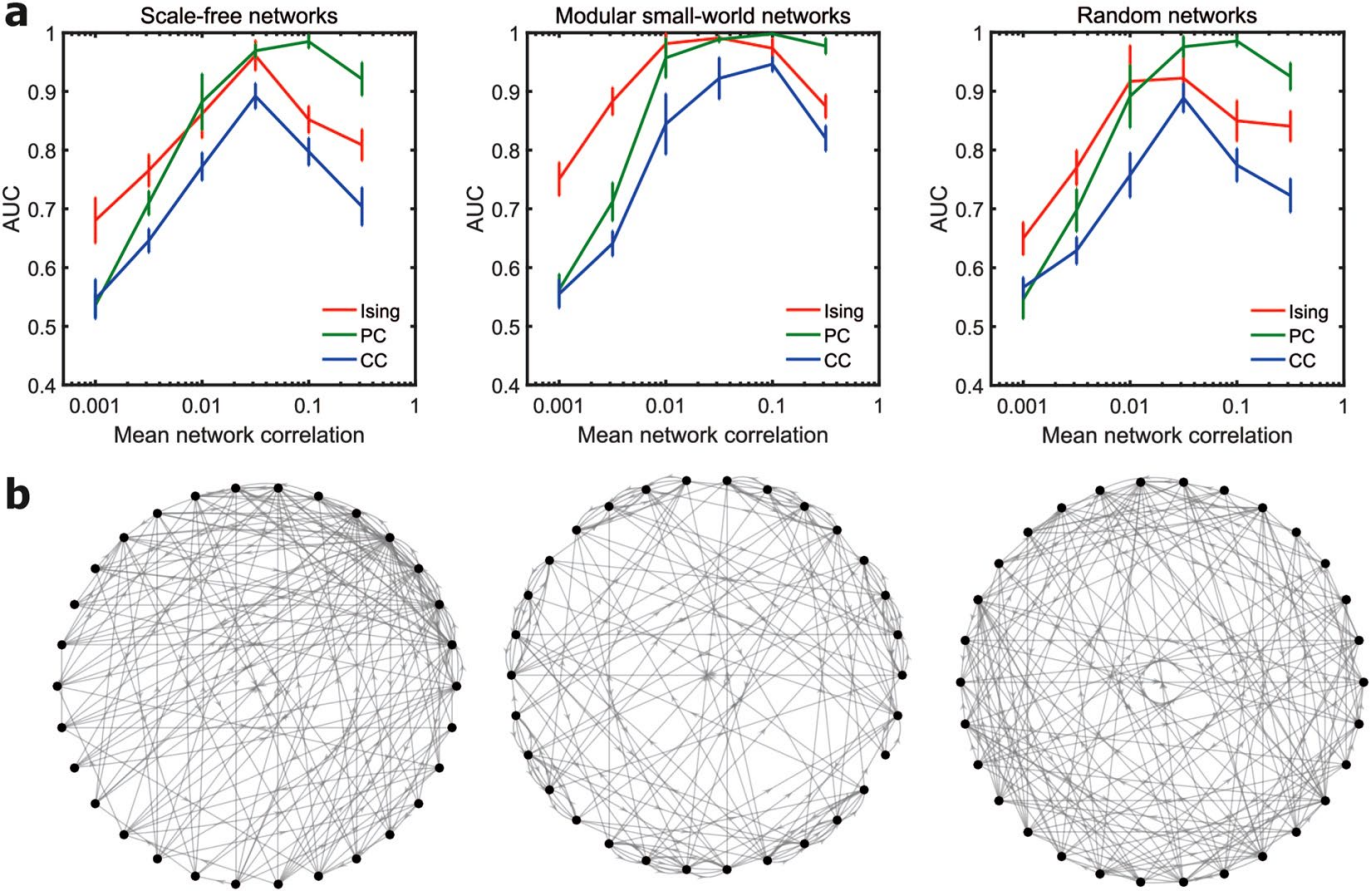
Effect of mean network correlation. **(a)** The first column in each row shows the raster plot of the spiking activity from a simulated neuronal network for a firing rate of 20 Hz and different network correlation levels. Histogram of the Ising couplings, partial correlations and cross-correlations for the pairs of neurons that are synaptically connected and not connected are shown respectively in the second, third and fourth columns. The corresponding ROC curves of the three functional connectivity metrics are shown in the last column. The first, second and third rows correspond to mean network correlation levels (*ρ*) of 0.001, 0.03 and 0.3 respectively. **(b)** Plot of the AUC values for different mean network correlation levels in scale-free networks of 30 neurons for a fixed firing rate of 20 Hz. Mean value was calculated from ten simulated networks. For weaker correlation levels (0.001 and 0.003), AUC value of Ising couplings was significantly higher than partial and cross-correlations. For stronger correlation levels (0.1 and 0.3), partial correlations had a significantly higher AUC value compared to Ising couplings and cross-correlations (*p* $<$ 0.01, two-sample t-tests). **(c)** True positive rate (TPR) and false positive rate (FPR) for the reconstruction of the structural connections by the three functional connectivity metrics thresholded at a sparsity threshold value of 20%.

We initially studied the performance of the three functional connectivity measures to uncover the underlying synaptic connectivity for different levels of network correlation for fixed firing rates in scale-free networks. The results for scale-free networks of 30 nodes for a fixed mean firing rate of 20 Hz is shown in Fig. 1b. For very weak levels of correlation (*ρ* = 0.001 and *ρ* = 0.003), partial correlations and cross-correlations performed no better than a random classifier and their AUC values were close to 0.5. In contrast, the AUC value of Ising couplings was significantly higher when compared to partial correlations and cross-correlations at very weak levels of correlation (*p *< 0.01, two-sample t-tests). When the network correlation level increased upto a value of 0.03, the AUC of Ising couplings increased and then gradually decreased. This can be explained as follows. When the network correlation was small, the synaptic connectivity in the network had a very weak effect on the spike trains of the neurons in the network and the neurons with the weakest synaptic connections were indistinguishable from the unconnected neurons. As the correlation level increased, the effect of synaptic connectivity on the spike trains became stronger, and the gap between the correlation in the spike trains of the connected neurons and the unconnected neurons increased. As a direct result, the detectability of the links also increased. However, after a particular point, the effect of the indirect connections became stronger, and it became difficult for Ising couplings to distinguish between the direct connections and the indirect connections and the AUC dropped as a result.

The AUC curve of partial correlations followed a similar pattern. However, the performance of partial correlation increased at a much faster rate with increase in the correlation levels, and soon it equalled and eventually surpassed Ising couplings for strong levels of correlation (*ρ* = 0.1 and *ρ* = 0.3). The AUC of partial correlations was significantly higher than that of Ising couplings for strong levels of correlation (*p* < 0.01, two-sample t-tests). For intermediate levels of network correlation (*ρ* = 0.01 and *ρ* =0.03), there was no difference between the AUC values of Ising couplings and partial correlations. The superior performance of the partial correlations at stronger levels of network correlations can be explained as follows. When the network correlation is strong, a spike in the presynaptic neuron evokes a spike in the postsynaptic neuron with high probability, and a linear dependency emerges between the spike train of the presynaptic and the postsynaptic neurons. Though indirect interactions emerge in the case of strong network correlations, the relationship between the spike trains of the indirectly connected neurons is still linear. As partial correlation can remove the linear effects of the population, partial correlations were able to discount the effect of spurious indirect interactions introduced at stronger levels of network correlation. We tested for the range of correlation levels for different fixed firing rates and different network sizes and found that the same trend persisted for all cases.

The AUC score gives a good summary of the performance of the functional connectivity metrics for every possible threshold value. However, in practice, we have to use a single threshold value typically. We tested the quality of reconstruction of the structural links for a sparsity threshold value of 20% (the strongest 20% of the functional connectivity links are considered to represent the structural links) based on the prior knowledge of the network density of the structural network, and the results are presented in Fig. 1c. The results are in general agreement with the results obtained earlier using the AUC scores. We can see that a higher AUC score in Fig. 1b corresponds to a higher true positive rate (TPR) and a lower false positive rate (FPR) in Fig. 1c.

### Effect of mean firing rate

**Figure 2:**
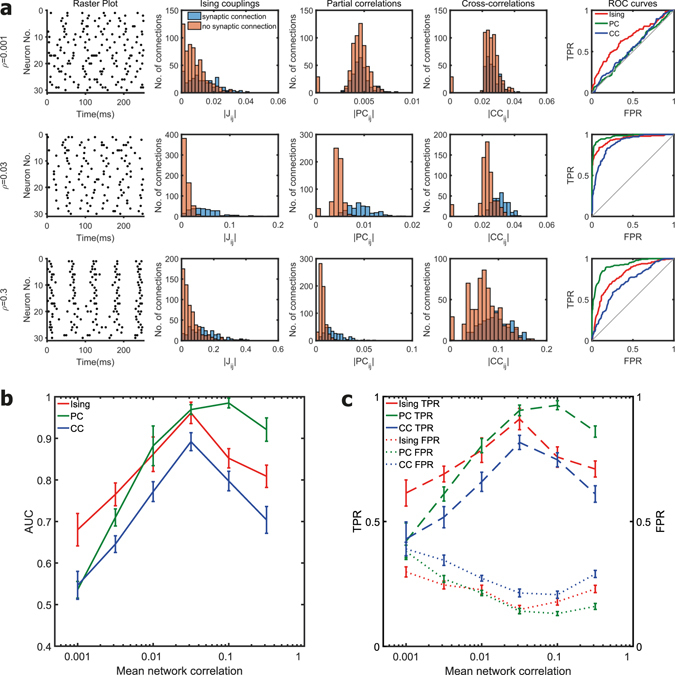
Effect of mean network firing rate. (**a)** The first and second rows correspond to firing rates of 10 Hz and 40 Hz respectively for a fixed correlation level (*ρ*) of 0.001. The third and fourth rows correspond to firing rates of 10 Hz and 40 Hz respectively for a fixed correlation level of 0.3. Raster plot of the spiking activity from a simulated neuronal network is shown in the first column. Histogram of the Ising couplings, partial correlations and cross-correlations for the pairs of neurons that are synaptically connected and not connected are shown respectively in the second, third and fourth columns. The corresponding ROC curves of the three functional connectivity metrics are shown in the last column. **(b)** and **(c)** Plot of the AUC values for different firing rates in scale-free networks of 30 neurons for fixed mean network correlation levels of 0.001 and 0.3 respectively. Mean value was calculated from ten simulated networks.

We then studied the effect of mean firing rate on the quality of recovery of the structural connections. Figures 2b and 2c present the effect of firing rate on the AUC of Ising couplings, partial and cross-correlations for fixed network correlation levels of 0.001 and 0.3, respectively. At a weak correlation level of 0.001, the AUC values of partial and cross-correlations remained low at around 0.5 and the AUC of Ising couplings was significantly higher than those of partial and cross-correlations for all firing rates (*p* < 0.01, two-sample t-tests). At a strong value of correlation of 0.3, all the three functional connectivity metrics show an increase in performance with an increase in firing rates. The relative difference between the AUC scores of partial correlations and Ising couplings persisted, and partial correlation detected significantly (*p* < 0.01, two-sample t-tests) more links when the correlation was strong for all the firing rates considered. Our observation that the AUC of partial correlations and cross-correlations increases with firing rates is consistent with the similar observations of Eichler^10^.

### Effect of network topology

**Figure 3:**
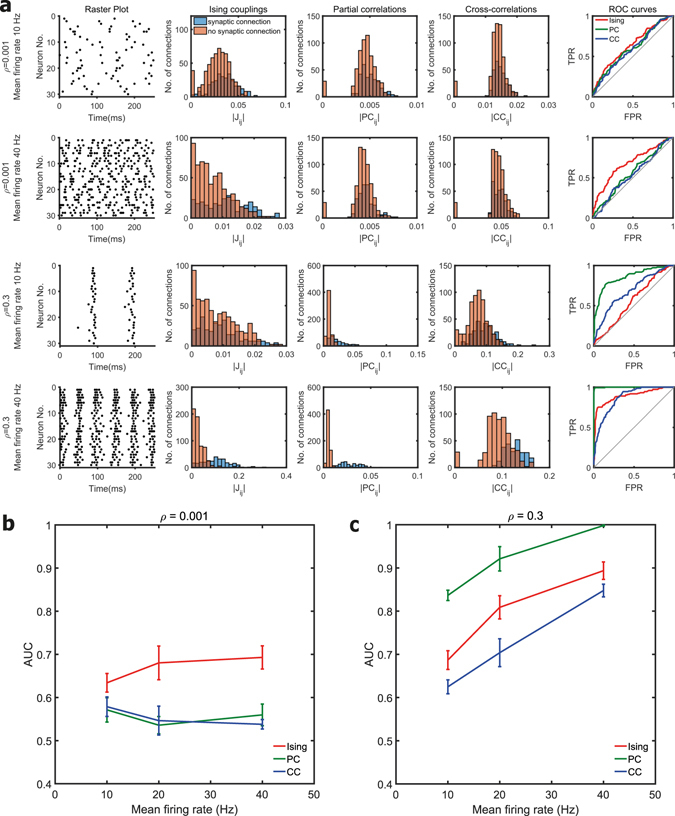
Effect of network topology. **(a)** Plot of the AUC values for networks of 30 neurons with scale-free (SF), small-world (SW) and Erdos-Renyi (ER) random network topologies. Data was averaged over ten simulated networks for each network condition. Firing rate was fixed at 20 Hz in all cases. All the three topologies had the same link density of 0.2. **(b)** An example of the structural connectivity network for each topology. Scale-free networks form a few highly connected hub nodes. Modular small-world networks present a balance of segregation and integration via dense intra-module connections and sparse inter-module connections. Most nodes in random networks have a degree in the vicinity of the average degree of the network.

Apart from networks with scale-free connectivity, we assessed the performance of Ising couplings, cross-correlations and partial correlations in networks of neurons with modular small-world connectivity and random connectivity. We maintained the same link density across the three topologies. The results of the assessment for networks of 30 nodes for a mean firing rate of 20 Hz are plotted in Fig. 3. We observed that trend of how the AUC scores of the three functional connectivity metrics vary with the network correlation levels did not change across topologies. We also observed that the AUC scores of Ising couplings, partial and cross-correlations in scale-free topology were not considerably different from their corresponding scores in random topology. However, the AUC scores of the three metrics in the small-world networks were considerably higher than their corresponding scores in scale-free networks. The high relative performance of the metrics in the case of small-world networks when compared to scale-free networks or randomly connected networks can be explained as a direct effect of our topology construction. The modular small-world networks were constructed by linking together fully connected modules with randomly distributed inter-module connections^31^. The number of inter-module connections was fewer when compared to intra-module connections. Hence, each node was influenced more strongly by the direct interactions from the other nodes in the same module (there were no indirect interactions within a module as each node was connected to every other node in the module) when compared to the indirect interactions from nodes in the other modules. So, the effect of indirect interactions was weaker in the case of small-world networks when compared to scale-free and random networks. As a result, all the three functional connectivity metrics performed better at disentangling direct interactions from indirect interactions in the modular small-world topology when compared to the other two topologies. To sum up, we observed that Ising couplings performed better at weaker levels of correlations and partial correlations performed better at stronger levels of correlation irrespective of the underlying structural connectivity topology.

### Effect of network size

**Figure 4:**
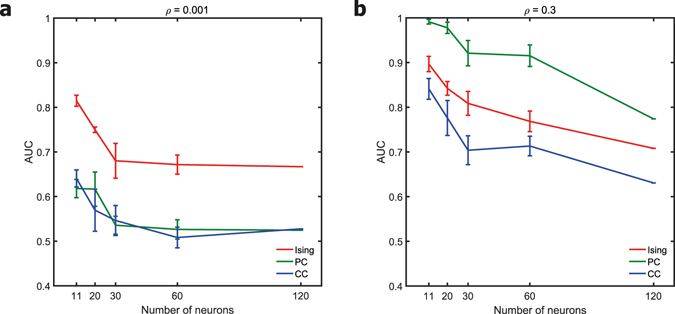
Effect of network size. Plot of the AUC values for networks of various sizes for a fixed firing rate of 20 Hz and correlation levels of 0.001 and 0.3 are displayed in the left panel **(**a**)** and the right panel **(**b**)** respectively. The mean value was calculated from ten networks for all cases except for networks of 120 nodes in which case the data is from the simulation of a single network.

We computed the three functional connectivity metrics for scale-free networks of different sizes (11, 20, 30, 60 and 120 nodes) and analysed how the network size affected the reconstruction of the structural connections. For both weak (Fig. 4a) and strong (Fig. 4b) correlation cases, all the three functional connectivity metrics displayed a reduced performance with an increase in the number of nodes. Partial correlation is known to have a reduction in performance with increased network size because of the so-called ‘marrying-parents’^10^ or ‘married-nodes’^13^

effect (When two neurons A and B share a postsynaptic neuron C, then the two input neurons A and B can become correlated as an artifact). Our results show that Ising model also suffers a reduction in detectability of the structural links for larger networks. Though the performance of all the three metrics decreased with increase in network size, the relative performance difference between Ising model and partial correlation remained. As a result, Ising couplings had the highest AUC in weaker correlation levels in networks of all sizes and partial correlations was the winner at stronger correlation levels in networks of all sizes.

### Effect of network density

**Figure 5:**
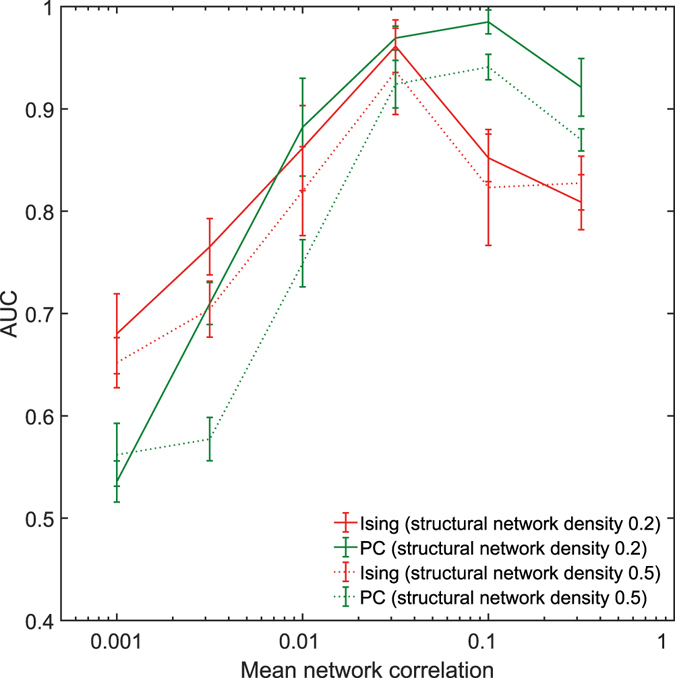
Effect of network density. Plot of the AUC values of Ising couplings and partial correlations for two different network densities and a fixed firing rate of 20 Hz and varying correlation levels. Data was averaged over ten scale-free networks of 30 nodes.

The structural networks considered so far had a network density of 0.2. To study the impact of the network density, structural networks were constructed with a network density of 0.5. The new structural networks were simulated to generate activity patterns, and the resulting functional connectivity metrics were computed. Figure 5 shows the plot of the AUC scores for Ising couplings and partial correlations for networks with the network density 0.2 and 0.5. It can be observed that even for networks with a higher network density of 0.5, the pattern of Ising couplings performing better at lower values of correlation and partial correlations performing better at higher values of correlation was preserved. Another interesting observation is that the AUC score of partial correlations in networks with higher network density is significantly smaller when compared to the corresponding scores in networks with a network density of 0.2 (*p* < 0.01, two-sample t-tests). This observation is consistent with the similar observations of Poli *et al*.^13^. The reduced performance of the partial couplings with increasing network density can again be attributed to the marrying-parents effect. Ising couplings also showed a reduced performance when the network density increased. However, the study did not find any significant statistical difference between the AUC scores of Ising couplings corresponding to the networks with two different network densities (*p* < 0.01, two-sample t-tests).

### Impact of the fit of Ising parameters

**Figure 6:**
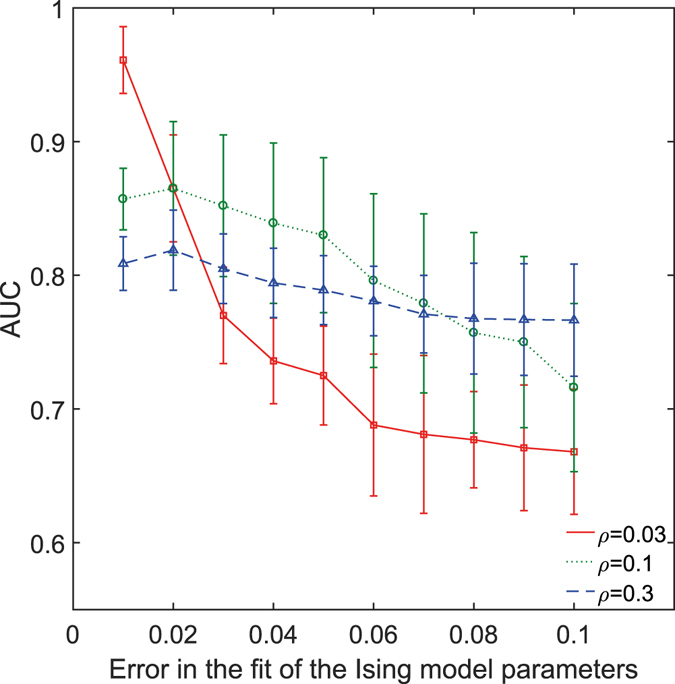
Effect of fit of the Ising model parameters on the inference of structural links. The error in the fit of the Ising model parameters is plotted against the AUC values obtained for the corresponding error levels for three mean network correlation levels (*ρ*) and a fixed firing rate for scale-free networks of 30 neurons. In all cases, lower the error in the fit of the Ising model parameters, higher was the detection of links in the structural connectivity matrix.

We computed the Ising parameters using the gradient descent method. We defined the cost function as the maximum difference between the 〈σ_*i*_〉 or $\langle {\sigma }_{i}{\sigma }_{j}\rangle$ of the Ising model and that of the data from the simulation. The cost function quantified the error in the fit of the Ising model parameters. Greater the difference between the averages of the model and the data, greater is the error in the fit of the model parameters. We ran the gradient descent algorithm for different values of the cost function and studied how the accuracy with which we fitted the Ising model parameters affected the reconstruction of the structural connectivity. Figure 6 shows the plot of the AUC values for different values of the error in the fit of the Ising model parameters. It can be seen that the capability of the Ising parameters to reconstruct the structural connectivity (given by the AUC score) increased with the decrease in the error in the fit of model parameters. It is to be noted that the gradient descent algorithm takes more time to compute more accurate model parameters. Thus, the number of the structural links correctly detected by the Ising parameters depends on the accuracy of the estimation of the model parameters, which, in turn, depends on the time the gradient descent algorithm is run for. In comparison, partial and cross-correlations can be computed using analytical solutions and also the time required to compute partial correlations is a fraction of the time required to compute Ising parameters, especially for larger systems.

## Discussion

Functional connectivity metrics have been widely used to infer the underlying structural connectivity of the neuronal circuits^8,22,39^. However, the conventional functional connectivity metric of cross-correlation is susceptible to the impact of indirect interactions arising out of poly-synaptic connections and common inputs. Maximum entropy based Ising models have been suggested to be able to discount the effect of indirect interactions and to account for only the direct ones^16,19,20^. Similarly, partial correlation approach has also been reported to remove the linear contribution of other neurons in the population and measure the direct interaction strength^10,12^. Which of the above two functional connectivity approaches provides the best measure of the underlying structural connectivity remains an open question, which has been addressed in this paper.

We found that the relative performance of the three functional connectivity tools was determined primarily by the network correlation levels (Fig. 1). Partial and cross-correlations performed only as well as a random classifier at very weak levels of network correlation (*ρ* = 0.001). In contrast, Ising couplings had a considerably higher AUC score when compared to partial correlations when the correlation levels were very weak (*ρ* = 0.001 and *ρ* = 0.003). However, partial correlation gained the advantage when the network correlation increased and performed better than Ising couplings at higher correlation levels (*ρ* = 0.1 and *ρ* = 0.3). At higher network correlation levels, whenever a presynaptic neuron spikes, there is a high chance that the postsynaptic neuron will spike as well and the relation between the spike trains of the neurons in the network tend to become linear. As partial correlations can remove the linear effects of the activity of all other neurons while assessing the relationship between two spike trains^12^, partial correlations outperform Ising couplings at higher network correlation levels. We found that the trend was consistent across different firing rates, network sizes and network topologies. Studies on networks of vertebrate retina^40,41^ have reported that the correlation between the activity of pairs of neurons is usually very weak (correlation coefficients in the range 0.001 to 0.1). This encourages further studies applying Ising models to assess structure-function relationship for *in vivo* and *in vitro* networks of neurons at low correlations. At the same time, partial correlations are a better choice in networks with high levels of correlation.

With technological advances^42,43^, the number of electrodes on the MEA are on the rise and the performance of the functional connectivity metrics for larger network size becomes important. It is known that the AUC of partial correlations will deteriorate when the number of neurons in the network increases because of the ‘married-nodes’ effect^10^. We observed that the AUC of Ising couplings also decreased when the number of neurons increased. Though all the three functional connectivity metrics suffered a reduction in AUC with an increase in the number of neurons in the network (Fig. 4), the relative AUC score amongst the three tools did not vary with the network size. Ising couplings had the highest AUC at weaker correlation levels, and partial correlations had the highest AUC at stronger correlation levels for all tested network sizes.

In addition to considerations of the accuracy of estimating the structural connectivity, the choice of the appropriate tool should also be informed by other factors such as speed of computation. The time required to compute the functional connectivity metrics becomes an important consideration, particularly for larger networks. The Boltzmann learning method used to calculate the Ising parameters is a very slow gradient descent algorithm^34^. For a larger number of nodes, we also have to run long Monte Carlo sampling steps per iteration as an exact computation of the moments of the Ising model are computationally expensive^35^. In comparison, analytical solutions exist to compute partial and cross-correlations in a much shorter span of time. For example, computation of partial and cross-correlations for a network of 60 neurons took in the order of minutes using the ToolConnect toolbox^37^ whereas the computation of Ising couplings using the Boltzmann learning method took in the order of hours (on the same hardware and under similar load conditions). Faster approximation methods^34,44^ exist to compute Ising couplings quickly. Each approximation method makes a few assumptions about the structure of the underlying network and firing conditions. When the assumptions are not fully met, it will affect the fit of the model parameters. We, therefore, studied how the accuracy of the fit of the Ising model parameters affected the reconstruction of the underlying structural connectivity matrix (Fig. 6). We observed that the smaller the error in the fit of the model parameters, the higher was the detection of the links in the structural connectivity matrix. This effect has to be taken into account when opting for a quick approximate solution vs. a time consuming exact solution to compute Ising parameters.

Roudi *et al*.^34^ calculated equilibrium Ising coupling parameters for a simulated model of cortical network and found no significant relation between the Ising couplings and the synaptic connectivity of the network. The poor performance of the equilibrium Ising model in their work could be attributed to the symmetry of its undirectional couplings, which were nevertheless used to estimate the asymmetric directional connections of the simulated network. For meaningful comparison and analysis between the structural and functional connectivity matrices, both matrices should be reduced to a sparse binary undirected form, through thresholding, binarising, and symmetrising^31^. The significantly improved results we obtained for Ising couplings corroborate this approach for comparison between the structural and functional connectivity matrices.

There have been enhancements to the standard Ising model. When Glauber dynamics is added to the model, it is referred to as kinetic Ising model^45^. The couplings of the kinetic Ising model are asymmetric and can account for the directionality of the links. Hertz *et al*.^46^ observed that the couplings of a kinetic Ising model are successful in recovering the synaptic connectivity of a simulated cortical network when compared to a standard Ising model. Our objective here has been to study the capability of standard Ising model couplings under different conditions in comparison to the partial and cross-correlations. Hertz’s results might be taken to indicate that neural system’s state transitions are described by the temporal dynamics of the stochastic process. However, in spite of the fact that neural systems might indeed be non-equilibrium, our results may indicate that the systems we investigated in this paper were to a large extent governed by equilibrium states, which can be described by equilibrium Ising models. It is worth noting that Ising model itself will not apply to systems far from equilibrium. A similar study of the capabilities of kinetic Ising model couplings in comparison to partial correlation and other functional connectivity measures for networks involving both excitatory and inhibitory neurons under different network conditions will be one of the avenues for future research.

## Conclusion

In summary, we performed a systematic study to benchmark the performance of Ising couplings to reconstruct the underlying structural connections in comparison to partial and cross-correlations, in *in silico* neuronal networks. We assessed the effect of firing rate, network correlation, network size, network density, and topology on the performance of the three functional connectivity metrics. This paper presents the key finding that the relative performance of the three functional connectivity tools depended primarily on the network correlation levels. Amongst the three compared functional connectivity metrics, Ising couplings detected the most structural links at weaker correlation levels and partial correlations at stronger correlation levels. These results were consistent across various firing rates, network sizes, and topologies. All the three functional connectivity measures showed a decreased detectability of the structural links with an increase in the number of neurons in the network. Understanding the strengths and weaknesses of the individual functional connectivity metrics, the network conditions in which they are applied and the computational time demands should serve as a guide in choosing the right functional connectivity tool to reconstruct the structural network topology.
