# Publisher Correction: Inferring structural connectivity using Ising couplings in models of neuronal networks

**DOI:** 10.1038/s41598-018-22950-1

**Published:** 2018-03-14

**Authors:** Balasundaram Kadirvelu, Yoshikatsu Hayashi, Slawomir J. Nasuto

**Affiliations:** Brain Embodiment Lab, Biomedical Engineering, School of Biological Sciences, University of Reading, Reading, United Kingdom

Correction to: *Scientific Reports* 10.1038/s41598-017-05462-2, published online 15 August 2017

The HTML version of this Article contains an error in the order of the Figures. Figures 1, 2 and 3 were published as Figures 2, 3 and 1 respectively. The correct Figures appear below. The Figure legends are correct. The PDF version was correct at the time of publication.

**Figure 1:**
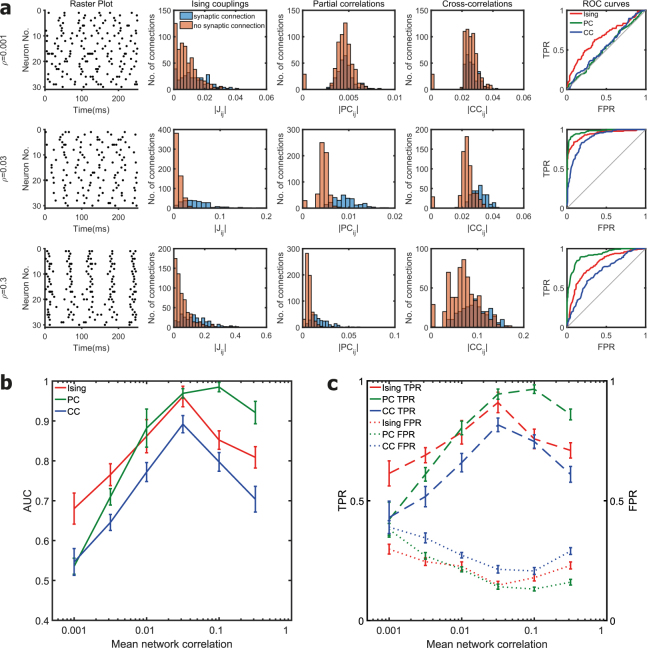
Effect of mean network correlation. **(a)** The first column in each row shows the raster plot of the spiking activity from a simulated neuronal network for a firing rate of 20 Hz and different network correlation levels. Histogram of the Ising couplings, partial correlations and cross-correlations for the pairs of neurons that are synaptically connected and not connected are shown respectively in the second, third and fourth columns. The corresponding ROC curves of the three functional connectivity metrics are shown in the last column. The first, second and third rows correspond to mean network correlation levels (*ρ*) of 0.001, 0.03 and 0.3 respectively. **(b)** Plot of the AUC values for different mean network correlation levels in scale-free networks of 30 neurons for a fixed firing rate of 20 Hz. Mean value was calculated from ten simulated networks. For weaker correlation levels (0.001 and 0.003), AUC value of Ising couplings was significantly higher than partial and cross-correlations. For stronger correlation levels (0.1 and 0.3), partial correlations had a significantly higher AUC value compared to Ising couplings and cross-correlations (*p* < 0.01, two-sample t-tests). **(c)** True positive rate (TPR) and false positive rate (FPR) for the reconstruction of the structural connections by the three functional connectivity metrics thresholded at a sparsity threshold value of 20%.

**Figure 2:**
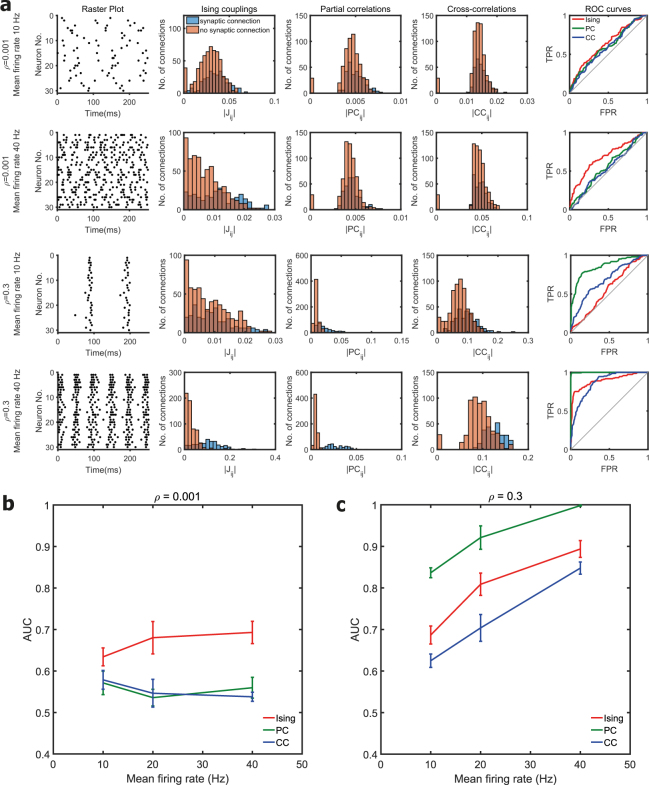
Effect of mean network firing rate. (**a)** The first and second rows correspond to firing rates of 10 Hz and 40 Hz respectively for a fixed correlation level (*ρ*) of 0.001. The third and fourth rows correspond to firing rates of 10 Hz and 40 Hz respectively for a fixed correlation level of 0.3. Raster plot of the spiking activity from a simulated neuronal network is shown in the first column. Histogram of the Ising couplings, partial correlations and cross-correlations for the pairs of neurons that are synaptically connected and not connected are shown respectively in the second, third and fourth columns. The corresponding ROC curves of the three functional connectivity metrics are shown in the last column. **(b)** and **(c)** Plot of the AUC values for different firing rates in scale-free networks of 30 neurons for fixed mean network correlation levels of 0.001 and 0.3 respectively. Mean value was calculated from ten simulated networks.

**Figure 3:**
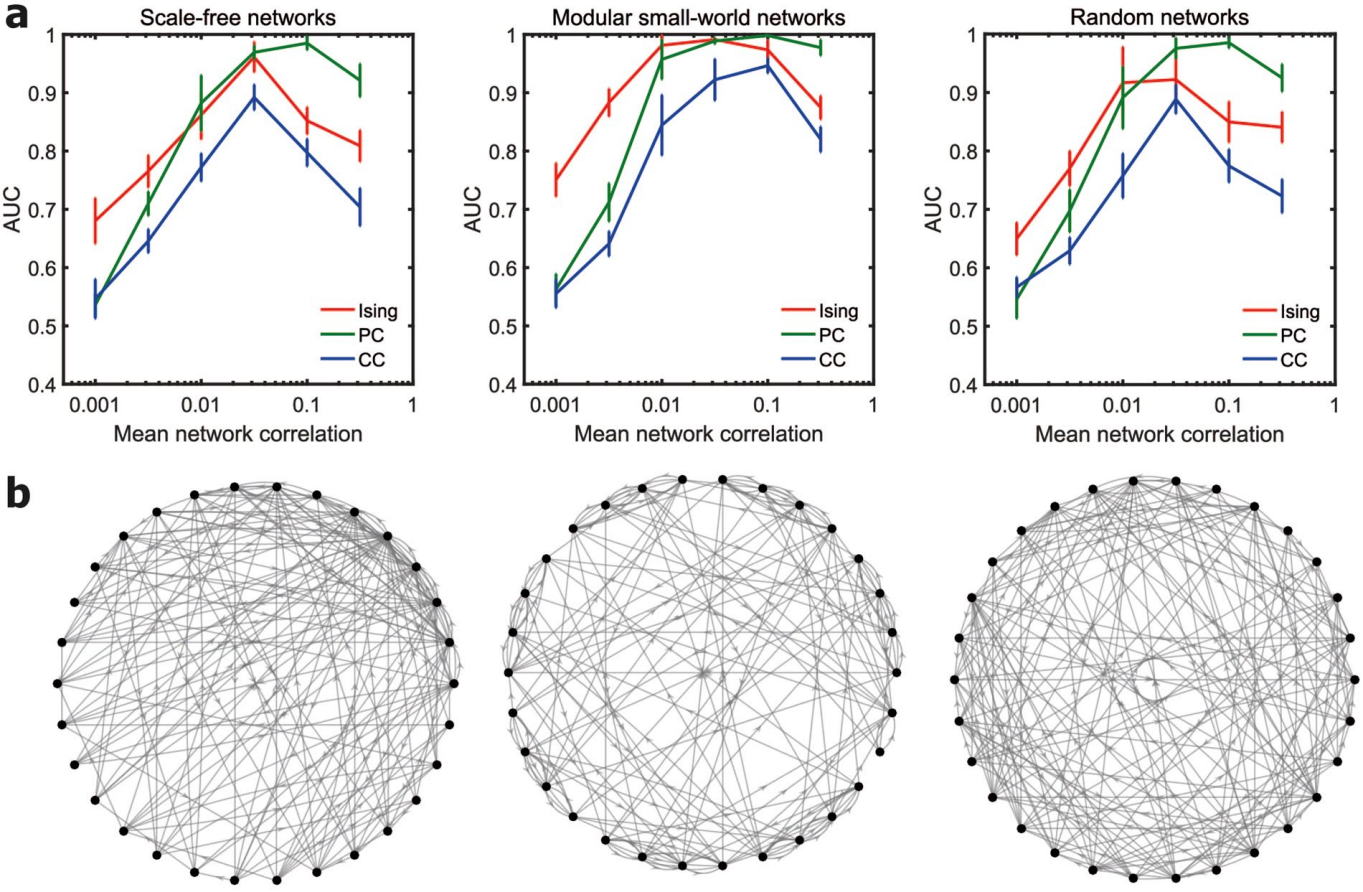
Effect of network topology. **(a)** Plot of the AUC values for networks of 30 neurons with scale-free (SF), small-world (SW) and Erdos-Renyi (ER) random network topologies. Data was averaged over ten simulated networks for each network condition. Firing rate was fixed at 20 Hz in all cases. All the three topologies had the same link density of 0.2. **(b)** An example of the structural connectivity network for each topology. Scale-free networks form a few highly connected hub nodes. Modular small-world networks present a balance of segregation and integration via dense intra-module connections and sparse inter-module connections. Most nodes in random networks have a degree in the vicinity of the average degree of the network.

